# Supplementary material for: ‘All We Have to Decide Is What to Do with the Time That Is Given to Us’ a Photovoice Study on Physical Activity in Nursing Homes
Source: Int J Environ Res Public Health. 2021 May 20;18(10):5481. doi: 10.3390/ijerph18105481 (PMC8160610; doi:10.3390/ijerph18105481)
Supplement: Supplementary file 1 [file ijerph-18-05481-s001.zip › ijerph-1209952-supplementary.pdf]

Englisch	Deutsch
Long stretches without obstacles promote physical activity, as they can be used by residents with wheelchairs and walkers regardless of weather conditions	lange Wege ohne Hindernisse sind fördernd, da auch für Rollstühle und Rollatoren wetterunabhängig geeignet“
Closed doors are impedimental because the desire for physical activity is halted. "[residents]you shall not pass"	″Verschlossene Türen hemmen da Bewegungsdrang unterbrochen wird. „Sie kommen nicht weiter“
Obstacles inhibit movement and increase the risk of falling; it is difficult to get past them	Gegenstände im Weg sind hemmend und Sturzgefahr, man kommt schlecht daran vorbei“
Not enough space is restrictive for PA, there is no space to turn around with a wheelchair or a rollator	zu wenig Platz ist hemmend kein Platz zum Wenden mit Rollstuhl und Rollator, ortgebunden“
The resident is tied to the place	Bewohnende sind durch Platzmangel ortsgebunden
Staircases are beneficial for PA, as [their use] results in the activation of more muscle groups (contracture prophylaxis), independence from the elevator and the opportunity to move about the entire house.	Treppen sind bewegungsförderlich, da mehr Muskelgruppen beansprucht werden → Kontrakturprophylaxe, Unabhängigkeit vom Aufzug, Bewegung im ganzen Haus möglich“ „. Verlassen des Hauses nur mit Hilfe möglich“
If the staircases cannot be negotiated, mobility is significantly restricted	Wenn die Treppe nicht überwunden werden kann, wird der Bewegungsradius deutlich eingeschränkt
A defective elevator inhibits PA because movement is restricted to the living area under certain circumstances	Defekter Aufzug hemmend, da Bewegungsradius unter Umständen auf ein Wohnbereich begrenzt ist
In the interior courtyard residents with and without dementia [residents] can walk around outside independently	Im Innenhof können sich auch demente Bewohnende frei bewegen
Standing on their legs with their full body weight	Stehen mit ganzem Gewicht auf den Beinen
Being able to stand up straight	Aufrechtes stehen möglich
The device performs the task of getting up, not muscular strength	Gerät übernimmt das Ausstehen nicht Muskelkraft
[residents] Need to use their arms and hands to grip and hold	Müssen Arme und Hände bewegen zum festhalten
Walkers are beneficial as they reduce the risk of falling, are an alternative to manual fixation, rests are possible at any time	Walker sind förderlich, da Verringerung der Sturzgefahr, Alternative zur Fixierung, Pausen jederzeit möglich
Rollators are supportive because they provide a feeling of security, thus interrupting the fear-of-falling spiral, rests are possible, objects can be transported in the basket, thus allowing to focus on walking and not on carrying	Rollatoren sind förderlich weil, gibt Sicherheit, dadurch Durchbrechen der Angst- Sturz Spirale, Pausen sind möglich, Dinge können im Korb transportiert werden, dadurch Konzentration auf gehen und nicht aufs tragen
The wheelchair promotes the ability to move with one's hands and feet, to overcome greater distances, rests are possible at any time	Rollstuhl fördert, weil Möglichkeit mit Händen und Füßen sich fortzubewegen, auch größere Distanzen zu überwinden, Pausen jederzeit möglich
The wheelchair is restrictive [for physical activity], because you let yourself be pushed, you become lazier and more comfortable	Rollstuhl ist hemmend, weil man sich schieben lässt, fauler und bequemer wird
Footrests inhibit PA because residents have no possibility to move by themselves and cannot move their feet and legs	Fußstützen sind bewegungshemmend weil Bewohnende keine Möglichkeit zur eigenen Bewegung haben, können die Füße und Beine nicht bewegen.
Footrests promote PA in hemiparesis, leg prostheses, one leg on the footrest the other is moving	Fußstützen sind bewegungsförderlich bei Hemiparesen, Beinprothesen, ein Bein auf der Fußstütze das andere wird bewegt.
Flower care promotes PA, if flowers are not cared for, they will die and residents have to take care of them, no matter how they are doing	Blumenpflege fördert da, wenn Blumen nicht versorgt werden gehen sie kaputt man muss sich um sie kümmern, egal wie das eigene Befinden ist.

Familiar activities from the past (which can be conveyed/transferred to dementia), harvesting (e.g. of herbs or tomatoes) gives a sense of success which enhances the motivation to succeed	Bekannte Tätigkeiten von früher (auch für Demente umsetzbar), Ernte (z.B. von Kräutern oder Tomaten) geben ein Erfolgserlebnis Motivation
music = distraction from physical handicaps	Musik = Ablenkung von körperlichen Gebrechen,
You can also move to music while sitting, you can't move wrongly to music, it is often associated with positive memories / experiences, enhances motivation.	Bewegung zu Musik auch im Sitzen möglich ist, zu Musik gibt es keine falsche Bewegung, wird oft mit positiven Erinnerungen/Erlebnissen in Verbindung gebracht, Motivation
Animals are allowed in the nursing home and are beneficial for the residents – provide many stimuli for PA	Tiere sind im Heim erlaubt und förderlich für die Bewohnenden- geben viele Anreize
Escape from everyday life and distracts and [let them] forget their frailties	Clowns im Dienst<br>Ausbruch vom Alltag, Ablenkung und Vergessen der Gebrechlichkeiten
Children encourage PA because they [residents] want to prove something, distraction by children, forgetting limitations	Kinder fördern, weil etwas beweisen wollen/zeigen dass man auch noch was kann, Ablenkung durch Kinder, Vergessen von Einschränkungen
